# Metabolomic Response of Equine Skeletal Muscle to Acute Fatiguing Exercise and Training

**DOI:** 10.3389/fphys.2020.00110

**Published:** 2020-02-18

**Authors:** Dylan J. Klein, Kenneth H. McKeever, Emily T. Mirek, Tracy G. Anthony

**Affiliations:** ^1^Department of Health and Exercise Science, Rowan University, Glassboro, NJ, United States; ^2^Rutgers Equine Science Center, Department of Animal Sciences, Rutgers, The State University of New Jersey, New Brunswick, NJ, United States; ^3^Department of Nutritional Sciences, Rutgers, The State University of New Jersey, New Brunswick, NJ, United States; ^4^New Jersey Institute for Food, Nutrition, and Health, Rutgers, The State University of New Jersey, New Brunswick, NJ, United States

**Keywords:** equine, skeletal muscle, metabolomics, gene expression, exercise, muscle metabolome

## Abstract

The athletic horse, despite being over 50% muscle mass, remains understudied with regard to the effects of exercise and training on skeletal muscle metabolism. To begin to address this knowledge gap, we employed an untargeted metabolomics approach to characterize the exercise-induced and fitness-related changes in the skeletal muscle of eight unconditioned Standardbred horses (four male, four female) before and after a 12-week training period. Before training, unconditioned horses showed a high degree of individual variation in the skeletal muscle metabolome, resulting in very few differences basally and at 3 and 24 h after acute fatiguing exercise. Training did not alter body composition but did improve maximal aerobic and running capacities (*p* < 0.05), and significantly altered the skeletal muscle metabolome (*p* < 0.05, *q* < 0.1). While sex independently influenced body composition and distance run following training (*p* < 0.05), sex did not affect the skeletal muscle metabolome. Exercise-induced metabolomic alterations (*p* < 0.05, *q* < 0.1) largely centered on the branched-chain amino acids (BCAA), xenobiotics, and a variety of lipid and nucleotide-related metabolites, particularly in the conditioned state. Further, training increased (*p* < 0.05, *q* < 0.1) the relative abundance of almost every identified lipid species, and this was accompanied by increased plasma BCAAs (*p* < 0.0005), phenylalanine (*p* = 0.01), and tyrosine (*p* < 0.02). Acute exercise in the conditioned state decreased (*p* < 0.05, *q* < 0.1) the relative abundance of almost all lipid-related species in skeletal muscle by 24 h post-exercise, whereas plasma amino acids remained unaltered. These changes occurred alongside increased muscle gene expression (*p* < 0.05) related to lipid uptake (*Cd36*) and lipid (*Cpt1b*) and BCAA (*Bckdk*) utilization. This work suggests that metabolites related to amino acid, lipid, nucleotide and xenobiotic metabolism play pivotal roles in the response of equine skeletal muscle to vigorous exercise and training. Use of these and future data sets could be used to track the impact of training and fitness on equine health and may lead to novel predictors and/or diagnostic biomarkers.

## Introduction

The horse is supremely muscular and athletic and is therefore highly dependent upon skeletal muscle metabolism for locomotion, racing performance, and overall health. To date, most studies in equine skeletal muscle have centered on a limited and targeted number of metabolites during acute exercise or training (Snow and Mackenzie, 1977; Snow et al., 1985; Harris et al., 1991; Jang et al., 2017). These observations have typically been related to energy production and utilization (e.g., lactate, pyruvate, creatine phosphate) and have been constrained to within minutes after the cessation of acute exercise (Schuback et al., 1999; McGowan et al., 2002). As such, less attention has been paid to global changes in skeletal muscle metabolism with acute exercise and training, as well as the early (3 h) and late (24 h) recovery periods and the metabolic alterations that take place during these crucial time points. With the arrival of “-*omics*” technologies, it is now possible to identify hundreds of metabolites in a biological sample that can serve to profile the metabolic response of skeletal muscle to acute exercise and training (Heaney et al., 2017; Liu and Locasale, 2017). Currently, the horse is one of the least-studied of the livestock animals with regard to metabolomics-based analyses (Goldansaz et al., 2017), especially in skeletal muscle (Jang et al., 2017). A better understanding of the exercise-induced metabolomic responses in skeletal muscle can (i) help to elucidate fitness-related metabolic signatures and the putative mechanisms that govern the beneficial adaptations of skeletal muscle to acute exercise and training, and (ii) provide much-needed information about the equine skeletal muscle metabolome, as very little data exists at this point in time.

Therefore, we aimed to characterize the exercise-induced and fitness-related changes in the equine skeletal muscle metabolome in eight unconditioned Standardbred horses before and after a 12-week training period. Using a non-targeted metabolomics approach, it was hypothesized that acute fatiguing exercise and 12 weeks of training would alter the exercise-induced and basal skeletal muscle metabolomic signatures, respectively. Further, it was hypothesized that training status would modify the metabolomic response to acute fatiguing exercise.

## Materials and Methods

### Animals

The Rutgers University Institutional Animal Care and Facilities Committee approved all methods and procedures used in this experiment. Eight rested and unconditioned Standardbred horses (*n* = 4 mares, *n* = 4 geldings; 3–8 years old) were used in this study (Table 1). All horses were weighed and underwent a physical examination to establish suitability for inclusion within the study. All of the horses were seen by a veterinarian and were deemed healthy and free of metabolic syndrome or any other endocrine disease that might impact metabolism or the response to exercise and training. All horses were dewormed and vaccinated per standard veterinary practice. Each horse was acclimated to the Rutgers University Equine Exercise Physiology Lab personnel, housing, and training equipment in the 2 months prior to the start of the experiment. Animals were fed a maintenance ration (∼1% of body mass, calculated on a dry matter basis) of alfalfa/grass hay *ad libitum* (∼6 kg/d) and a high-protein (14% CP) pelleted grain concentrate (∼3 kg/d; Brown’s Feeds 14% Ultra Horse Pellet) throughout the study period (DE ∼17 Mcals/d). Water and mineral blocks were also provided *ad libitum*. The horses were housed, by sex, in groups of four per two-acre dry lot paddock.

**TABLE 1:** Physiological and performance characteristics before and after 12-weeks of training.

	Unconditioned		Conditioned	
		
Variable	Geldings (*n* = 4)	Mares (*n* = 4)	Geldings (*n* = 4)	Mares (*n* = 4)
Body mass (kg)	479.8 ± 53.3	489.3 ± 26.3	464 ± 44	495 ± 18
Rump fat thickness (cm)	1.4 ± 0.1	2.1 ± 0.5	1.0 ± 0.1	2.2 ± 0.6^#^
Body fat (%)	10.4 ± 0.5	13.9 ± 2.9	8.1 ± 0.7	14.5 ± 3.3^#^
Fat-free mass (kg)	429.9 ± 47.9	420.8 ± 16.0	427 ± 40	423 ± 16
Fat mass (kg)	49.9 ± 6.1	68.4 ± 17.0	38 ± 5	72 ± 18^#^
VO_2__max_ (ml/kg/min)	153.8 ± 14.2	141.8 ± 17.6	167 ± 8*	158 ± 6*
GXT max velocity (m/s)	9.8 ± 0.5	9.3 ± 0.5	11 ± 0*	10.9 ± 0.5*
Total run time (s)	320 ± 31.4	290 ± 21.6	413 ± 15*	374 ± 15*^#^
Total distance (m)	2,243.8 ± 308.0	1,952.5 ± 197.5	3,218 ± 165*	3,004 ± 275*^#^

Data are presented as means ± SD. *p < 0.05, significantly different from before training; ^#^*p* < 0.05, significant difference between sexes.

### Experimental Design

**FIGURE 1:**
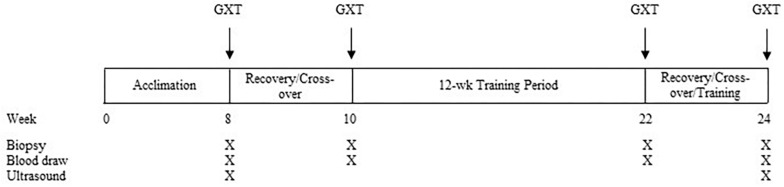
Experimental protocol timeline. Muscle biopsies and blood samples were taken approximately 30 min prior to GXT and at 3 and 24 h post-GXT. Following the training period, pre-exercise biopsies and blood draws were taken 72 h after the last exercise session.

Each horse completed the experimental protocol over a period of ∼24 weeks (Figure 1). In brief, the study had two exercise trials that were each comprised of a graded, incremental exercise test (GXT; described below), completed alongside time-matched, standing controls (SC). Sample collection (muscle biopsies and blood draws; described below) was completed before, during, and after each bout. Following a 2-month acclimation period, four horses performed an initial, pre-training GXT on a high-speed treadmill (Sato I, Lexington, KY, United States) while four horses served as SC. Following a 2-week washout period that allowed for healing of the muscle biopsies, in crossover fashion, horses that had completed the GXT then served as SC, and vice versa. Pre-training exercise testing was followed by 12 weeks of conditioning exercise, carried out 4 days/week in a motorized equine exercise machine (Equi-ciser, Calgary, Canada) with heavy exercise undertaken 1 day/week on the same high-speed treadmill. Seventy-two hours after the last exercise session of the 12-week training period, the same horses undertook a similar bout of post-training exercise, in crossover fashion, in the trained state. During the healing/cross-over period, horses were continually trained to maintain fitness status.

### Graded, Incremental Exercise Test (GXT)

The GXTs used previously published methods (McKeever and Malinowski, 1997; Kearns and McKeever, 2002) to measure maximal oxygen uptake (VO_2__max_) and indices of exercise performance. The mean room temperature of the lab during exercise was approximately ∼20°C. During the GXTs, the horses ran on a high-speed horse treadmill (Sato I, Lexington, KY, United States) at a fixed 6% grade and started at an initial speed of 4 m/s for 1 min. Speed was then increased to 6 m/s (omitting 5 m/s), followed by incremental increases of 1 m/s every 60 s until the horses reached fatigue. Fatigue was defined as the point at which the horse could no longer keep up with the treadmill speed despite humane encouragement. At the point of fatigue, the treadmill was stopped, the velocity and time to fatigue were recorded, and the horse was taken off and cooled down via hand walking. Following cool-down, horses were allowed water *ad libitum* and feed was withheld until after the 3 h biopsy.

### Oxygen Consumption and Carbon Dioxide Production Measurements

Oxygen consumption (VO_2_) and carbon dioxide production (VCO_2_) was measured every 10 s using an open-flow indirect calorimeter (Oxymax-XL, Columbus Instruments, Columbus, OH, United States) during each GXT, using previously described methods with reported coefficients of variation (CV) < 0.1 (McKeever and Malinowski, 1997).

### Training

Horses were randomly assigned, with an equal number of mares and geldings, to groups of four for the training period. This was done in order to stagger the training days and prevent running all eight horses on the treadmill on the same day (Table 2). Training began in April of 2015 and was concluded in July of the same year. Training was comprised of a standardized exercise procedure (SEP; Table 2) wherein exercise volume (time) and intensity (speed) was increased every week over the 12-week study period (Supplementary Table S1). For the heavy treadmill exercise, the galloping speed during the workout for each horse was increased each week up to a safe maximal intensity. This was determined both objectively (i.e., horses are capable of maintaining their running position on the treadmill with humane encouragement) and subjectively (e.g., horse handlers skilled observation of level of fatigue). The purpose of the conditioning period was to get all eight horses to a fitness level at which they would have oxygen consumption and carbon dioxide production values that are indicative of being in the trained state (∼160–170 ml/kg/min) (Hodgson and Foreman, 2014). Over the course of the study all horses were able to tolerate the exercise program and no injuries or side effects were documented (e.g., lameness).

**TABLE 2:** Schedule of exercise sessions during the 12-week training period*.

**Day of the week**	**Group A (2 mares, 2 geldings)**	**Group B (2 mares, 2 geldings)**
Sunday	Paddock (free-exercise)	Paddock (free-exercise)
Monday	Light exercise	Light exercise
Tuesday	Moderate exercise	Light exercise
Wednesday	Light exercise	Moderate exercise
Thursday	Heavy exercise	Light exercise
Friday	Light exercise	Heavy exercise
Saturday	Paddock (free-exercise)	Paddock (free-exercise)

*The full training program can be found in Supplementary Table S1. Light exercise – consisted of walking (2 m/s) and trot/canter (4 m/s). Moderate exercise – consisted of walking (2 m/s); trot/canter (4 m/s); and slow gallop (6 m/s). Heavy exercise – consisted of walking (2 m/s); GXT to fatigue; and a 5 min cool down via hand walking.

### Body Composition Measurements

Percent body fat was estimated before and after the training period using the prediction equation from Kane et al. (1987) where:% Body fat = 2.47 + 5.47 x (rump fat thickness in cm). Rump fat thickness (RFT) was directly measured using B-mode ultrasonography (Aloka SSD-500, Tokyo, Japan) by placing the probe over the rump approximately 5 cm lateral from the midline, at the center of the pelvic bone (Westervelt et al., 1976). The region was scanned and the position of maximal fat thickness was used as the measurement site. Scans were repeated in triplicate and the values were averaged. Fat mass (FM, kg) was calculated by multiplying% body fat and total body mass (TBM, kg). Fat-free mass (FFM, kg) was calculated as the difference between total body mass and fat mass (i.e., FFM = TBM – FM).

### Muscle Biopsy Sampling

Percutaneous needle biopsies were obtained from the *M.* gluteus medius as previously described (Lindholm and Piehl, 1974) under sterile techniques using a Bergstrom biopsy needle (6 mm) with suction applied via a 140 mL syringe following aseptic scrubbing and subcutaneous lidocaine administration (∼2 mL). Biopsies (∼500 mg) were taken halfway (∼15 cm) along an imaginary line drawn from the head of the *tuber coxae* to the tail head at a standardized depth between 60 and 80 mm. Samples were immediately weighed, placed in aluminum foil sachets, and immersed in liquid nitrogen until transferred for storage at −80°C. Pre and post-exercise muscle biopsies were taken from a single incision (using a No. 10 blade scalpel) before (∼30 min prior), and at 3 h following exercise with the direction of needle pointed ∼2–3 cm away from the previously sampled site. The 24 h biopsy was taken in the contralateral leg using the same techniques and site standardization. Following training, new incisions were made approximately 3–5 cm away from previous incisions to avoid sampling previously biopsied locations. Incisions were closed via sutures and horses were monitored daily to ensure adequate healing of the incision site. No visible side effects were documented during these times (e.g., hematoma, lameness or alteration in gait).

### RNA Isolation

RNA isolation was carried out using modifications of previously published methods (Wagner et al., 2013). In brief, ∼30–40 mg of powdered muscle tissue was homogenized in 1 mL of TRIzol reagent using a Polytron homogenizer for ∼10 s. Following homogenization on ice for ∼10 s, 100 μL of BAN phase separator was added directly to each sample and vortexed for ∼5 s. Samples were subsequently centrifuged at 10,000 × *g* for 10 min and the supernatant transferred to a new tube where total RNA was precipitated overnight at −20°C using an equi-volume of isopropanol. The resultant pellet was washed four times in 70% ethanol and re-suspended in 30 μL of RNAsecure^TM^ (Thermo Fisher Scientific). Genomic DNA was removed using DNase I treatment (Thermo Fisher Scientific). RNA purity was quantified using a NanoDrop^®^ 1000 spectrophotometer (Thermo Fisher Scientific) and only samples with 260/280 ratios > 1.8 (mean 1.9) were used for cDNA synthesis and further analysis. Samples were diluted with nuclease free water to 100 ng/μL.

### cDNA Synthesis and Reverse Transcription Quantitative Polymerase Chain Reaction (RT-qPCR)

Ten μl of total RNA was reverse transcribed into cDNA using a high-capacity cDNA reverse transcription kit (Thermo Fisher Scientific) as per manufacturer’s specifications. Reactions were carried out in a total volume of 20 μL (10 μL of 100 ng/μL RNA plus 2 μL buffer, 2 μL primers, 0.8 μL dNTPs, 1 μL multiscribe, and 4.2 μL nuclease free water). Resultant cDNA was then diluted in 380 μl nuclease free water and stored at −20°C until further analysis. Oligonucleotide primers for RT-qPCR were generated using Primer3 online software^1^ and sent off for commercial synthesis (Integrated DNA Technologies, Inc.) The primer sequences used were as follows: *Cd36* forward: 5′-ttttctacggacctggcttg, reverse: 5′-acagtcctggggtctgtctg; *Cpt1b* forward: 5′-ggtcgacttccagctcagtc, reverse: 5′-tgtccacgttgcagtaggag; *Plin2* forward: 5′–ctgcctcc-tttcaggaagtg, reverse: 5′-gtctcctggctggctgtatc; *Bckdk* forward: 5′–atcattggctgcaaccc-tac, reverse: 5′–ccaagaag-tagcggacaagc; *Fabp3* forward: 5′-catgaagtcaatcggtgtgg, reverse: 5′–aagtttgcc-tccatccagtg; *Acc2* forward: 5′–tgttcgagttcatggagcag, reverse: 5′–atttctgtgca-ggggtgttc; *Gapdh* forward: 5′-gagatcaagaaggtggtgaagc, reverse: 5′-catcgaaggtgga-agagtgg. Quantitative RT-PCR of targets was performed, in duplicate, using Power SYBR Green Master Mix (Thermo Fisher Scientific) and detected using the Onestep PCR machine (Applied Biosystems, Foster City, CA, United States). The standard curve method was used to estimate qPCR efficiency^2^ and target gene mRNA expression was normalized to *Gapdh* (housekeeping gene) as this has been shown to be stable in equine skeletal muscle with training (Eivers et al., 2010).

### Metabolomics Bioinformatics

Approximately 100 mg of frozen muscle biopsy samples from exercised horses was sent on dry ice to Metabolon^®^, Inc. for ultra-high performance liquid chromatography-tandem mass spectroscopy (UPLC-MS/MS) analysis using their DiscoveryHD4 comprehensive global metabolomics platform. Samples were prepared and analyzed as per the company’s protocol (Gall et al., 2010). For metabolite identification, retention time/index (RI), mass-to-charge ratio (*m*/*z*), and MS/MS spectra were compared against authenticated standards as part of a library that is curated and maintained by Metabolon^®^, Inc. (Gall et al., 2010).

### Plasma Amino Acid Quantification

Plasma branched-chain amino acid (BCAA; leucine, isoleucine and valine) and phenylalanine concentrations were quantified via high-performance liquid chromatography (HPLC). Plasma samples were removed of all lipids, proteins, and surfactants via adding 180 μL of 0.1% formic acid in methanol with 60 μL of plasma for 5 min and filtering through an Agilent Captiva column (Agilent Technologies, Santa Clara, CA, United States) via a 3 mL plunger. All samples (20 μL) were run alongside internal standards (5 dilutions from 45 to 900 pmol/μl) on an Agilent ZORBAX Eclipse Plus C18 column (2.1 × 100 mm, 1.8 μm, 1200 bar) and analyzed using OpenLab software (Agilent Technologies, Santa Clara, CA, United States).

### Statistical Analysis

Data are presented as means ± SD. For the metabolomics statistical analysis, any missing values that were assumed to be below detection limits were imputed with the compound minimum (minimum observed value). Statistical analysis of log-transformed data was performed by Metabolon^®^, Inc. using ‘R’ (R foundation)^3^, a freely available, open-source software package. One-way ANOVA contrasts and Welch’s Two Sample *t*-tests were used to identify biochemicals that differed significantly between experimental groups (e.g., unconditioned *vs.* conditioned and mares *vs.* geldings, respectively). Two-way mixed model ANOVA contrasts were used to identify biochemicals exhibiting significant interaction and main effects for experimental parameters of treatment and time. Significance was defined as a *p*-value < 0.05. An estimate of the false discovery rate (FDR, *q*-value) was calculated to take into account the multiple comparisons that occur during ‘omics’-based studies (Storey and Tibshirani, 2003). A *q*-value < 0.1 was used as an indication of high confidence in a statistically significant result. Baseline physiologic and performance characteristics between geldings and mares was analyzed via Student’s *t*-test. Effects of sex and training status on VO_2__max_, BM, FFM, FM, RFT, %BF, run time to fatigue, and work completed were analyzed via two-way ANOVA for repeated measures using SigmaStat 4.0 software (San Jose, CA, United States), and *post hoc* analyses were determined using Tukey’s test with an alpha value of 0.05. For gene expression and plasma amino acid statistical comparisons, differences between the unconditioned and trained states were analyzed via paired *t*-tests. Significance was set at *p* < 0.05.

## Results

### Physiological and Performance Characteristics of Horses Before and After Training

Horses’ body composition, aerobic capacities and running capacities, before and after the 12-week training period have been previously published (Klein et al., 2020) and are summarized in Table 1.

### Training Produces a Homogenous Metabolomic Response to Acute High-Intensity Fatiguing Exercise in Equine Skeletal Muscle

**FIGURE 2:**
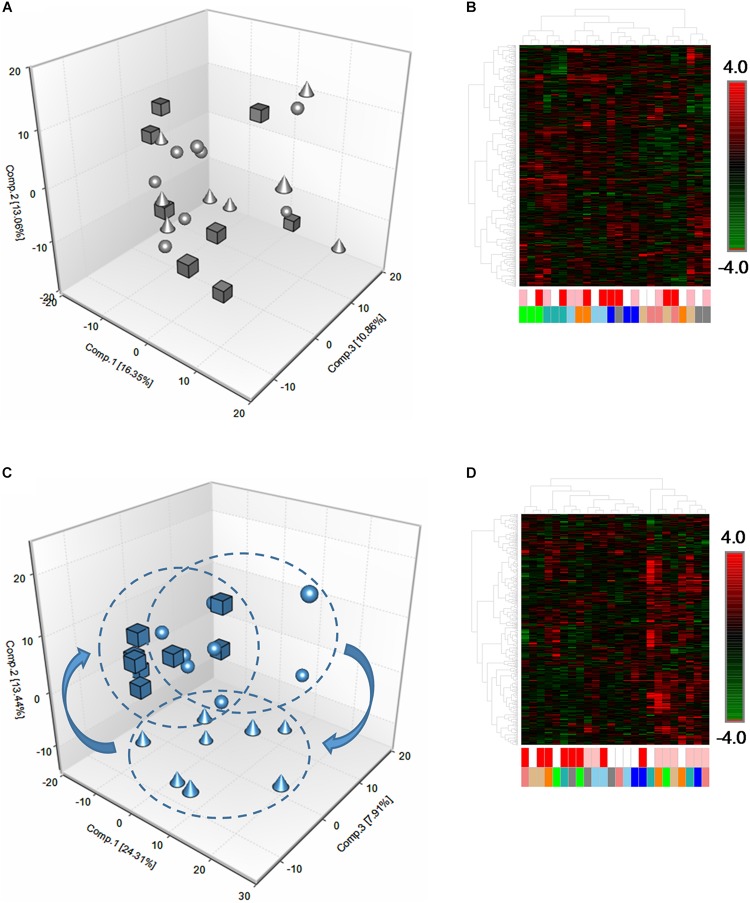
Global metabolomic responses to acute fatiguing exercise in equine skeletal muscle. **(A)** Three-dimensional PCA plot of skeletal muscle biopsy samples in the unconditioned state. Spheres denote pre-exercise (Pre), cones denote 3 h post-exercise (3 h), and cubes denote 24 h post-exercise (24 h). The plot reveals little discrimination between the time points with Component 1 (16.35%), Component 2 (13.06%), and Component 3 (10.86%). **(B)** Hierarchical clustering heatmap supports the findings from PCA plot (A) and revealed that samples originating from the same horse clustered close together rather than around a given time point. White boxes denote Pre, pink boxes denote 3 h, and red boxes denote 24 h. Green, blue, light blue, orange, gray, salmon, tan, and teal colors each signify an individual horse. **(C)** Three-dimensional PCA plot of skeletal muscle biopsy samples in the conditioned state. The plot reveals a better discrimination of horses between time points with Component 1 (24.31%), Component 2 (13.44%), and Component 3 (7.91%). **(D)** Hierarchical clustering heatmap of skeletal muscle biopsy samples in the conditioned state. Heatmap supports findings from PCA plot (C) and revealed that samples tended to cluster around a given time point rather than horses clustering with themselves. *n* = 8 per group.

In order to characterize the basal and exercise-induced metabolomic signatures of equine skeletal muscle, we employed a non-targeted, UPLC-MS/MS-based metabolomics approach in eight rested Standardbred horses (*n* = 4 geldings, *n* = 4 mares). Muscle samples were collected surrounding a bout of acute high-intensity fatiguing exercise before and after a 12-week training period. Metabolomic analysis revealed a total of 545 metabolites in the skeletal muscle samples. Principle Components Analysis (PCA), hierarchical clustering, and ANOVA contrasts were performed in order to ascertain the effects of high-intensity fatiguing exercise on skeletal muscle metabolomic profiles in the early (3 h) and late (24 h) recovery periods, as well as the degree to which individual horses differed from one another. Prior to training, in the unconditioned state, PCA revealed a high degree of heterogeneity among the individual skeletal muscle metabolomes, regardless of time point (Figure 2A). Hierarchical clustering (Figure 2B) also supported this finding showing that individual horses tended to cluster together rather than at a given time point.

In order to establish the effects of training on the global skeletal muscle metabolome before and after acute exercise, we carried out 12 weeks of aerobic training, interspersed with anaerobic training. PCA and hierarchical clustering of metabolomes showed a much greater homogeneity among the skeletal muscle samples, both basally, as well as after the bout of acute high-intensity fatiguing exercise (Figures 2C,D).

### Training Augments the Metabolomic Response in Skeletal Muscle Following Acute Fatiguing Exercise

Of the 545 metabolites identified in the skeletal muscle biopsy samples, only 31 were significantly altered (29 increased, 2 decreased; *p* < 0.05, *q* < 0.1) at 3 h post-exercise (Supplementary Table S2), with only 1 metabolite showing a significant change (increase, *p* < 0.05, *q* < 0.1) at 24 h in the unconditioned state (Supplementary Table S2). Further, only 12 metabolites (1 increased, 11 decreased) significantly differentiated the late (24 h) from early (3 h) recovery period (*p* < 0.05, *q* < 0.1; Supplementary Table S2).

In contrast, following training, there were 142 and 150 significantly altered metabolites at 3 h (100 increased, 42 decreased; *p* < 0.05, *q* < 0.1; Supplementary Table S2) and 24 h (13 increased, 137 decreased; Supplementary Table S2) post-exercise, respectively. Further, 223 metabolites distinguished the late (24 h) from the early (3 h) recovery period (26 increased, 197 decreased, *p* < 0.05, *q* < 0.1; Supplementary Table S2).

### Acute Exercise-Induced Metabolic Alterations Primarily Center on Amino Acid and Lipid Metabolism

Approximately half (14 out of 29) of the significantly altered (*p* < 0.05, *q* < 0.1) acute exercise-induced metabolite increases at 3 h in the unconditioned state centered on alterations in amino acid metabolism, particularly those related to branched-chain amino acid (BCAA) metabolism (6 out of 14) (Table 3). The remaining 15 compounds were associated with lipid (5), nucleotide (3), carbohydrate (3), vitamin/co-factor metabolism (1), energy metabolism (1), or were xenobiotic in origin (2) (Table 4). The two decreased metabolites were associated with carbohydrate and energy metabolism (ribose 1-phosphate and fumarate, respectively) (Table 3). Only *N*-methylalanine was significantly increased (1.4-fold, *p* < 0.05, *q* < 0.1) at 24 h in the unconditioned state.

**TABLE 3:** Amino acid-related metabolites significantly (*p* < 0.05, *q* < 0.1) altered 3 h post-exercise in the unconditioned state (*n* = 8).

**Metabolite name**	**Pathway**	Fold change (FC)*
*N*-methylalanine	Alanine and aspartate	3.58
4-methyl-2-oxopentanoate	BCAA	2.02
2-hydroxy-3-methylvalerate	BCAA	2.01
3-hydroxy-2-ethylproprionate	BCAA	1.85
3-methyl-2-oxobutyrate	BCAA	1.79
Alpha-hydroxyisovalerate	BCAA	1.75
Beta-hydroxyisovalerate	BCAA	1.39
Glutamine	Glutamate	1.26
Saccharopine	Lysine	3.62
2-aminoadipate	Lysine	3.38
2-hydroxybutyrate/	Methionine, cysteine, SAM	1.29
2-hydroxyisobutyrate	and taurine	
3-(4-hydroxyphenyl)lactate	Phenylalanine and tyrosine	1.79
Phenyllactate	Phenylalanine and tyrosine	1.50
Idolelactate	Tryptophan	1.81

*Fold change values normalized to pre-exercise time point.

**TABLE 4:** Lipid, nucleotide, carbohydrate, vitamin and co-factor, and xenobiotic-related metabolites significantly (*p* < 0.05; *q* < 0.1) altered 3 h post-exercise in the unconditioned state (*n* = 8).

**Metabolite name**	**Pathway**	Fold change (FC)*
Mannitol/sorbitol	Carbohydrate	1.82
Sedoheptulose	Carbohydrate	1.91
Ribose 1-phosphate	Carbohydrate	0.68
Fumarate	Energy	0.79
3-hydroxybutyrylcarnitine (1)	Lipid	1.64
3-hydroxybutyrylcarnitine (2)	Lipid	2.44
Arachidoylcarnitine (C20:0)	Lipid	4.46
2-hydroxyadipate	Lipid	1.98
*N*-palmitoyl-sphinganine (d18:0/16:0)	Lipid	1.90
Urate	Nucleotide	5.58
AICA ribonucleotide	Nucleotide	3.53
Xanthosine	Nucleotide	1.97
Quinolinate	Vitamin and co-factor	2.74
Methyl-4-hydroxybenzoate sulfate	Xenobiotic	3.56
Propyl-4-hydroxybenzoate sulfate	Xenobiotic	3.07

*Fold change values normalized to pre-exercise time point

In the conditioned state, the majority of significant (*p* < 0.05, *q* < 0.1) acute exercise-induced alterations in skeletal muscle metabolites at 3 h were similarly related to amino acid (37 increased, 7 decreased) and lipid metabolism (25 increased, 24 decreased) (Supplementary Table S4). This was followed by xenobiotic (14 increased), nucleotide (11 increased, 2 decreased), carbohydrate (10 increased, 2 decreased), and vitamin/co-factor metabolism (3 increased, 1 decreased) (Supplementary Table S4). At 24 h, most of the significant (*p* < 0.05, *q* < 0.1) exercise-induced alterations in metabolites were related to decreases in lipid, amino acid, and nucleotide-related compounds relative to 3 h post-exercise (Supplementary Table S4).

There was a conservation of 26 commonly affected metabolites in both the unconditioned and conditioned states at 3 h post-exercise (Table 5). Approximately half (12 out of 26) were related to amino acid metabolism. Conditioned muscle, however, exhibited an additional 74 significantly (*p* < 0.05, *q* < 0.1) increased metabolites at this time point, primarily those related to other lipid, amino acid, and nucleotide-related compounds (Supplementary Table S4). Likewise, the majority (41/45) of significantly (*p* < 0.05, *q* < 0.1) decreased metabolites at 24 h compared to 3 h in the unconditioned state (Supplementary Table S3) were also decreased in the conditioned state (Supplementary Table S4), with another 156 metabolites differentiating conditioned muscle from unconditioned muscle at this time point. Most of these alterations centered on decreases in additional lipid and amino acid-related compounds.

**TABLE 5:** Conservation of significantly (*p* < 0.05; *q* < 0.1) exercise-induced metabolite changes at 3 h post-exercise.

**Metabolite name**	**Pathway**	Fold change (FC)*	
		**UC (*n* = 8)**	**C (*n* = 8)**
*N*-methylalanine	Alanine and aspartate	3.58	1.80
4-methyl-2-oxopentanoate	BCAA	2.02	1.75
2-hydroxy-3-methylvalerate	BCAA	2.01	2.45
3-methyl-2-oxobutyrate	BCAA	1.79	1.99
Alpha-hydroxyisovalerate	BCAA	1.75	2.92
Beta-hydroxyisovalerate	BCAA	1.39	1.47
Saccharopine	Lysine	3.62	8.38
2-aminoadipate	Lysine	3.38	2.55
2-hydroxybutyrate/2-hydroxyisobutyrate	Methionine, cysteine, SAM and taurine	1.29	1.58
3-(4-hydroxyphenyl)lactate	Phenylalanine and tyrosine	1.79	2.15
Phenyllactate (PLA)	Phenylalanine and tyrosine	1.50	1.85
Indolelactate	Tryptophan	1.81	4.16
Mannitol/sorbitol	Carbohydrate	1.82	3.14
Sedoheptulose	Carbohydrate	1.91	4.18
Arachidoylcarnitine (C20)	Lipid	4.46	3.32
3-hydroxybutyrylcarnitine (2)	Lipid	2.44	2.47
3-hydroxybutyrylcarnitine (1)	Lipid	1.64	1.55
2-hydroxyadipate	Lipid	1.98	3.20
*N*-palmitoyl-sphinganine (d18:0/16:0)	Lipid	1.89	1.94
Urate	Nucleotide	5.58	10.72
AICA ribonucleotide	Nucleotide	3.53	6.93
Xanthosine	Nucleotide	1.97	2.51
Quinolinate	Vitamin	2.74	5.83
Methyl 4-hydroxybenzoate sulfate	Xenobiotic	3.56	13.71
Propyl 4-hydroxybenzoate sulfate	Xenobiotic	3.07	14.94
Quinate	Xenobiotic	2.20	1.87

*Fold change values are normalized to the pre-exercise time point for each respective group.

Based on Welch’s Two Sample *t*-test there were no significant differences in the metabolomic signatures between mares or geldings for any of the 545 identified metabolites (*p* > 0.05, *q* > 0.1). This includes their basal signatures as well as the metabolomic responses to exercise and training.

### Training Alters Nucleotide and Xenobiotic-Related Markers in Equine Skeletal Muscle

There was a significant (*p* < 0.05; *q* < 0.1) accumulation of certain nucleotide-related metabolites at both the pre-exercise and the 3 h time point in conditioned compared to unconditioned muscle (Table 6). Further, conditioned skeletal muscle exhibited a prominent xenobiotic-related signature compared to unconditioned muscle (Table 7). This was evidenced by an increase in the relative abundances of numerous xenobiotic and potentially xenobiotic metabolites pre and post-exercise.

**TABLE 6:** Significantly (*p* < 0.05; *q* < 0.01) altered nucleotide-related metabolites in the conditioned state (*n* = 8).

**Metabolite name**	Fold change (FC)*	
	**Pre-exercise**	**3 h post-exercise**
	**(*n* = 8)**	**(*n* = 8)**
AICA ribonucleotide	2.19	4.29
Inosine 5′-monophosphate (IMP)	0.61	0.59
Inosine	1.63	1.66
Hypoxanthine	1.58	1.56
Xanthine	1.47	1.69
Xanthosine	1.62	2.05
2′-deoxyinosine	2.09	2.29
Adenosine 5′-monophosphate (AMP)	1.39	0.85^NS^
Adenosine 3′-monophosphate (3′-AMP)	1.57	1.99
Adenosine 2′-monophosphate (2′-AMP)	1.62	1.95
Adenosine 3′,5′-cyclic monophosphate (cAMP)	1.70	1.26^NS^
Adenosine	1.89^NS^	3.34
Guanosine 5′-monophosphate (5′-GMP)	0.69	0.85^NS^
Guanosine	2.80	2.56
Uridine	1.27	1.48
Uracil	2.30	3.16
5-methyluridine (ribothymidine)	1.40	1.02^NS^
2′-deoxyuridine	1.38^NS^	3.09
3-ureidoproprionate	1.22	1.46

*Fold change values are normalized to unconditioned samples at the respective time point. NS, not a statistically significant fold change.

**TABLE 7:** Xenobiotic-related metabolic signature in conditioned skeletal muscle.

	Fold change (FC)*		
	
**Metabolite name**	**Pre-**	**3 h post-**	**24 h post-**
	**exercise**	**exercise**	**exercise**
	**(*n* = 8)**	**(*n* = 8)**	**(*n* = 8)**
Phenyllactate	1.24	1.52^#^	0.90
3-(4-hydroxy-phenyl)lactate	1.20	1.44^#^	1.00
Phenol sulfate	2.59^#^	2.79^#^	1.39
*p*-cresol-sulfate	1.85^#^	2.19^#^	1.81^#^
Indoleproprionate	0.92	1.75^#^	1.06
3-indoxyl sulfate	1.20	1.82^#^	0.86
4-ethylphenyl sulfate	5.84^#^	17.92^#^	9.72^#^
Methyl 4-hydroxy-benzoate sulfate	0.90	3.45^#^	0.36^#^
Propyl 4-hydroxy-benzoate sulfate	0.71	3.45^#^	0.56
Tartronate	0.96	2.44^#^	0.82

*Fold change values are normalized to unconditioned samples at the respective time point. ^#^*p* < 0.05; *q* < 0.1, significantly altered relative to the unconditioned state.

### Training Alters Lipid and Amino Acid Metabolism

**FIGURE 3:**
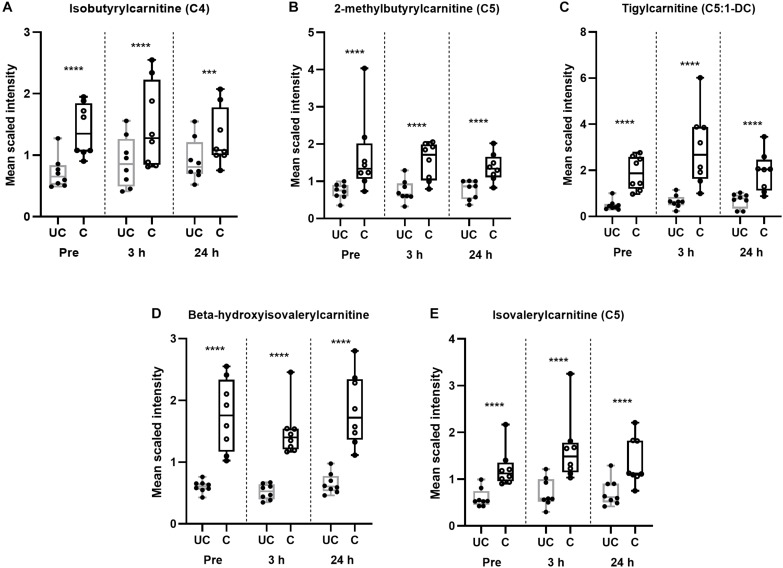
Training alters BCAA catabolism regardless of time point. **(A–E)** Box and whisker plots of BCAA-derived acylcarnitines in unconditioned (UC) and conditioned (C) skeletal muscle samples. Data are represented from minimum to maximum and show each individual point. The middle line of the box plot represents the mean. ^∗∗∗^*p* < 0.005, ^****^p < 0.0005, significant difference from UC, according to one-way ANOVA. *n* = 8 per group.

Twelve wks of training resulted in a significant (*p* < 0.05, *q* < 0.1) increase in almost every identified long-chain fatty acid (Table 8), as well as the accumulation of various other complex lipid species (e.g., phospholipids, sphingolipids, lysolipids, and lysoplasmalogens; Supplementary Table S5). Notably, there were also significant (*p* < 0.05, *q* < 0.1) elevations in intramuscular diacylglycerides (DAGs) and long-chain acylcarnitines (Table 9), as well as endocannabinoids (Supplementary Table S5). These complex lipid species have been shown to be elevated in muscle in individuals with obesity (Amati et al., 2011) and are implicated in causing insulin resistance (Itani et al., 2002; Heyman et al., 2012). Further, 12 weeks of training lead to significantly (*p* < 0.05, *q* < 0.1) increased abundances of BCAA-derived acylcarnitines (C4 and C5 species) at all time-points compared to the unconditioned state (Figures 3A–E). A number of these metabolites are also implicated in sedentary obesity in humans (Newgard et al., 2009).

**TABLE 8:** Elevation in long-chain fatty acids pre-exercise in the conditioned state (*n* = 8).

**Metabolite name**	Fold change (FC)*
Myristate (14:0)	1.40^#^
Pentadecanoate (15:0)	1.22^∧^
Palmitate (16:0)	1.48^#^
Palmitoleate (16:1 n-7)	1.40^∧^
Margarate (17:0)	1.50^#^
10-heptadecenoate (17:1 n-7)	1.34^∧^
Stearate (18:0)	1.31^#^
Oleate/vaccenate (18:1)	1.61^#^
Nonadecanoate (19:0)	1.37^∧^
10-nonadecenoate (19:1 n-9)	1.44^∧^
Arachidate (20:0)	1.20^∧^
Eicosenoate (20:1)	1.36^∧^
Erucate (22:1 n-9)	1.48^#^
Nervonate (24:1 n-9)	1.46^#^
Eicosapentaenoate (EPA; 20:5 n-3)	2.68^#^
Docosapentaenoate (n-3 DPA; 22:5 n-3)	1.86^#^
Docosahexaenoate (DHA; 22:6 n-3)	1.99^#^
Linoleate (18:2 n-6)	2.48^#^
Linolenate (18:3 n-3 or n-6)	2.14^#^
Dihomo-linolinate (20:3 n-3 or n-6)	2.05^#^
Arachidonate (20:4 n-6)	2.61^#^
n-6 DPA (22:5 n-6)	2.77^#^
Docosadienoate (22:2 n-6)	1.97^#^
Dihomo-linoleate (20:2 n-6)	2.02^#^
Mead acid (20:3 n-9)	3.03^#^

*Fold change values normalized to the pre-exercise time point in the unconditioned state. ^∧^0.05 < *p* < 0.1; *q* < 0.1, trend for statistical significance. ^#^*p* < 0.05; *q* < 0.1, statistically significant fold change relative to the unconditioned state.

**TABLE 9:** Significantly (*p* < 0.05; *q* < 0.1) elevated long-chain acylcarnitines and DAGs pre-exercise in the conditioned state (*n* = 8).

**Metabolite name**	**Pathway**	Fold change (FC)*
Laurylcarnitine (C12)	Long-chain acylcarnitine	2.16
Myristoylcarnitine (C14)	Long-chain acylcarnitine	1.93
Palmitoylcarnitine (C16)	Long-chain acylcarnitine	1.68
Stearoylcarnitine (C18)	Long-chain acylcarnitine	1.73
Linoleoylcarnitine (C18:2)	Long-chain acylcarnitine	1.75
Arachidonoylcarnitine (C20:4)	Long-chain acylcarnitine	1.99
Adrenoylcarnitine (C22:4)	Long-chain acylcarnitine	2.36
Dihomo-linolenoylcarnitine (C20:3 n-3 or n-6)	Long-chain acylcarnitine	1.86
Diacylglycerol (16:1/18:2 [2], 16:0/18:3 [1])	Diacylglyceride	1.77
Oleoyl-linoleoyl-glycerol (18:1/18:2) [1]	Diacylglyceride	1.55
Oleoyl-linoleoyl-glycerol (18:1/18:2) [2]	Diacylglyceride	1.59
Linoleoyl-arachidonoyl-glycerol (18:2/20:4) [2]	Diacylglyceride	1.87
Linoleoyl-linolenoyl-glycerol (18:2/18:3) [2]	Diacylglyceride	1.99
Palmitoyl-arachidonoyl-glycerol (16:0/20:4) [2]	Diacylglyceride	1.34
Palmitoyl-linoleoyl-glycerol (16:0/18:2) [1]	Diacylglyceride	1.93
Palmitoyl-linoleoyl-glycerol (16:0/18:2) [2]	Diacylglyceride	1.77
Palmitoyl-linolenoyl-glycerol (16:0/18:3) [2]	Diacylglyceride	1.61
Stearoyl-linoleoyl-glycerol (18:0/18:2) [2]	Diacylglyceride	1.52

*Fold change normalized to the pre-exercise time point in the unconditioned state.

**FIGURE 4:**
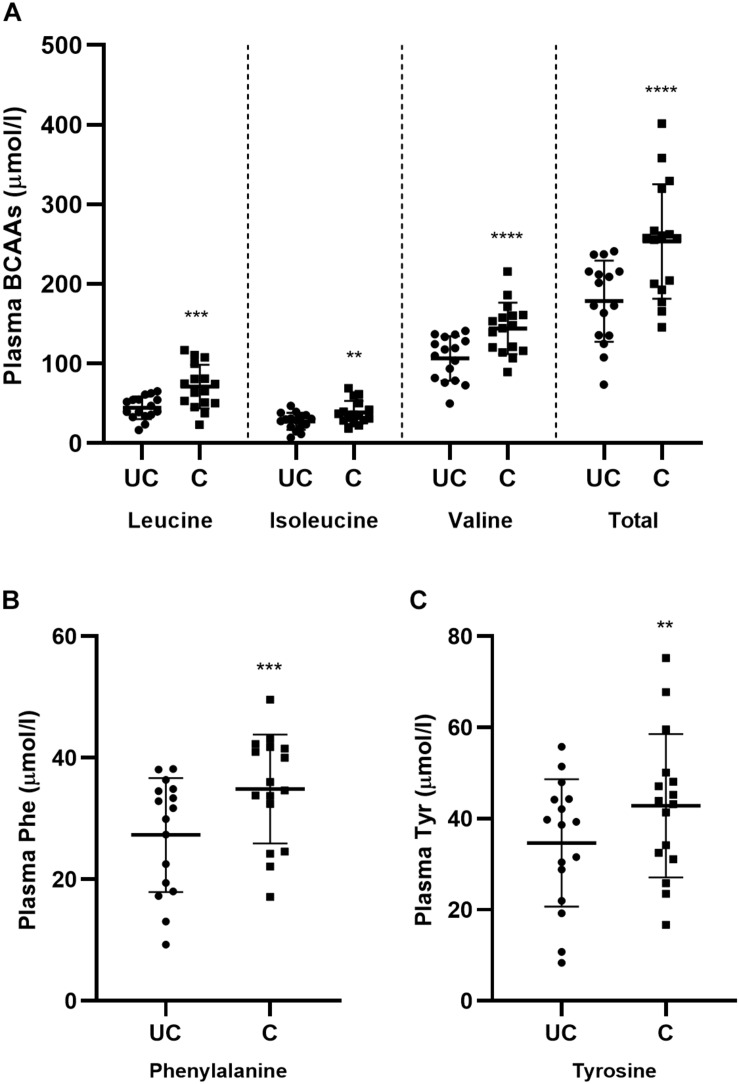
Training alters circulating plasma amino acid concentrations. **(A–C)** Scatter plots (mean ± SD) of all pre-exercise plasma BCAAs **(A)**, phenylalanine **(B)**, and tyrosine **(C)** in unconditioned (UC) and conditioned (C) horses. Samples from exercise and standing control days were pooled, *n* = 16 UC, *n* = 16 C. ^∗∗^*p* < 0.02, ^∗∗∗^*p* = 0.01, ^****^p < 0.0005, according to paired *t*-test.

Obesity and insulin resistance are have also been shown to be correlated with an increase in plasma concentrations of the BCAAs and the aromatic amino acids, phenylalanine and tyrosine (Felig et al., 1969; Newgard et al., 2009; Zhao et al., 2016). As such, we also measured these parameters in blood samples taken before and after the training period to determine the extent to which this signature is recapitulated in the circulation of conditioned horses. Interestingly, it was shown that training significantly (*p* < 0.05) increased the concentrations of the BCAAs, phenylalanine, and tyrosine (Figures 4A–C). Consequently, a muscle and blood metabolic signature previously shown to be associated with sedentary obesity in humans is manifest in conditioned horses following a 12-week training period that significantly improves aerobic capacity and exercise tolerance.

**FIGURE 5:**
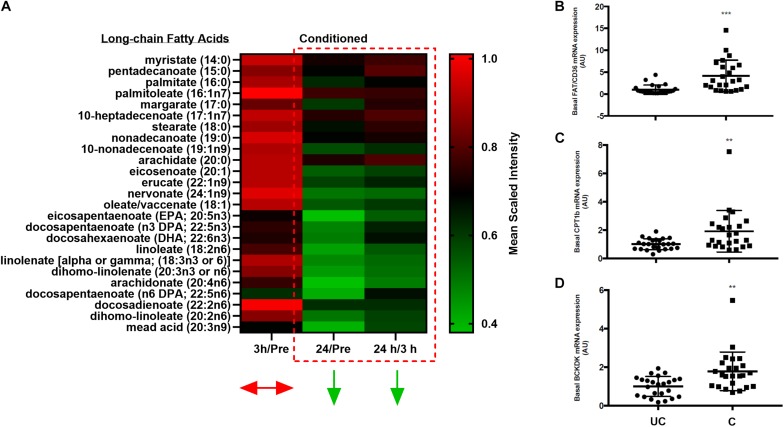
Changes in skeletal muscle free-fatty acid abundance and gene expression. **(A)** Heatmap depicting changes in the relative abundances of free-fatty acids over time in conditioned skeletal muscle. Color scale reflects mean scaled intensities. **(B–D)** Scatter plots (mean ± SD) of all pre-exercise and standing control skeletal muscle basal mRNA expression levels (pooled) of FAT/CD36 **(B)**, CPT1B **(C)**, and BCKDK **(D)** in unconditioned (UC) and conditioned (C) horses. *n* = 24 UC, *n* = 24 C. **p = 0.005, ***p = 0.0005, significant difference from UC, according to paired *t*-test.

One postulated mechanism that differentiates muscle insulin sensitivity between obese individuals and elite athletes is that athletes have greater muscle turnover of potentially lipotoxic intermediates (Goodpaster et al., 2001; Dube et al., 2008; Goodpaster and Sparks, 2017). As such, metabolically flexible individuals can utilize intramuscular fuels to a greater degree, thereby preventing their build-up and insulin-disrupting functions (Goodpaster et al., 2001). Herein, we showed that following even a brief (∼7–8 min) bout of high-intensity fatiguing exercise, the relative abundances of most lipid species in skeletal muscle were significantly (*p* < 0.05, *q* < 0.1) reduced by 24 h post-exercise (Figure 5A). Further, as aforementioned, BCAA catabolic intermediates are increased in skeletal muscle following exercise in the conditioned state (Figures 3A–E), suggestive of increased BCAA breakdown and utilization. This, however, was not mirrored in changes in plasma BCAA concentrations at 3 or 24 h post-exercise (data not shown).

### Training Alters Skeletal Muscle Gene Expression Related to BCAA Metabolism and Fatty Acid Uptake and Utilization

Given the above findings, we then explored gene expression changes that could be indicative of the alterations seen in BCAA and lipid metabolism in skeletal muscle with training. In this regard, training significantly (*p* < 0.05) increased the basal mRNA expression levels of genes related to fatty acid uptake (*Cd36*) and oxidation (*Cpt1b*), the regulation of BCAA breakdown (*Bckdk*), as well as a directional decrease in gene expression related to intramuscular lipid storage (*Plin2*, *p* = 0.08, data not shown) (Figures 5B–D). No meaningful change in the expression of other candidate genes was noted (e.g., *Acc2*; data not shown).

## Discussion

Given its large skeletal muscle mass and innate ability to exercise at high intensities, the horse positions itself as a unique model organism to understand the effects of exercise on muscle metabolism. Previous investigations in the horse have aimed to characterize skeletal muscle gene and protein expression patterns that occur with acute exercise and training (McGivney et al., 2009, 2010; Bouwman et al., 2010), but none, to the best of our knowledge, have been conducted regarding global metabolomic responses that are downstream of transcriptional and proteomic processes. The present findings demonstrate that an untargeted metabolomic investigation of equine skeletal muscle affords the opportunity to understand broad skeletal muscle metabolic changes in response to acute exercise and training, with most alterations centering on changes in amino acid, lipid, nucleotide and xenobiotic-related metabolites. Given the dearth of literature regarding the equine skeletal muscle metabolome, we hope that future investigations extend upon these novel findings.

To the best of our knowledge, this is the first report using an untargeted metabolomics approach and has found reduced skeletal muscle metabolomic heterogeneity with improved training status in mammals. From a transcriptional perspective, it has been shown in one study that highly trained cyclists have reduced interindividual differences in basal muscle gene expression relative to untrained controls (Wittwer et al., 2004). Coupled with the present data, this suggests that chronic exercise stimuli can potentially attenuate regulatory molecular differences that are apparent in the unconditioned state. Nevertheless, biological factors such as the variability of fiber type distribution within muscle (Elder et al., 1982), as well as the potential for greater muscle fiber homogeneity with improved training status (Rodriguez et al., 2002) must also be considered.

To this end, it has been shown in the horse that the variability of fiber types taken from serial *gluteus medius* biopsies is small (Lopez-Rivero et al., 1991), and that samples taken from the deeper regions of the *gluteus medius* are more homogenous in fiber size than more superficial regions (Lopez-Rivero et al., 1992). Given that all the biopsies in the current study were rigorously standardized and taken from a similar location, and from the deeper region of the *gluteus medius*, it is unlikely that these factors contributed significantly to the metabolomic profiles. Similarly, it has been shown that the populations of muscle fiber types do not significantly change in Standardbreds with approximately 12 weeks of endurance training (Hodgson et al., 1986; Tyler et al., 1998) despite changes in capillary density, mitochondrial volume, and oxidative capacity (Hodgson et al., 1986; Tyler et al., 1998). As such, the present data suggests that chronic exercise, that significantly improves aerobic and running capacities, dominates the skeletal muscle phenotype and produces a more homogenous metabolic profile, both basally and in response to acute exercise. Future studies designed specifically to address this question, however, are strongly advised in order to confirm or clarify this interpretation.

Most of the metabolomic patterns or signatures centered on changes in the conditioned state, with a greater magnitude of amino acid and lipid-related metabolites being altered post-exercise relative to the unconditioned state. Horses rely heavily on muscle glycogen for high intensity exercise (Lacombe et al., 2003), however, they can require up to 72 h post-exercise to fully replete their muscle glycogen stores (Waller and Lindinger, 2010). Interestingly, in the present study, we did not show a significant alteration in glycogen-related metabolites post-exercise in the unconditioned state (Supplementary Table S3), but did observe a significant reduction in two (of four) glycogen-related metabolites at 3 h post-exercise in the conditioned state (Supplementary Table S4). This suggests a greater reliance on glycogen for the post-training acute exercise bout, which is not surprising given the greater work volume completed (Table 1). By 24 h, however, these metabolites were fully recovered which suggests that the exercise bout may not have been substantially glycogen depleting. This is not unreasonable given that most glycogen-depleting protocols (of >50%) in horses have utilized multiple high-intensity sprints to fatigue (Lacombe et al., 2001) or much longer duration, lower-intensity exercise (Snow et al., 1981). A notable limitation of the present study is that we did not directly measure glycogen levels, and future studies should aim to measure this metabolite in order to place global metabolomic patterns in the full context of post-exercise muscle energy status.

Interestingly, there was a conservation of 26 metabolites that were altered 3 h post-exercise, regardless of training status. Many of these are related to amino acid metabolism, particularly BCAA catabolism. In humans and rodents it is known that the BCAAs can be utilized in skeletal muscle for energy production during acute, submaximal endurance exercise (White and Brooks, 1981; Wolfe et al., 1982; Wagenmakers et al., 1989). Currently it is unclear whether or not horses similarly use BCAAs in skeletal muscle during exercise (Poso et al., 1991). The present data suggest that amino acids, particularly the BCAAs, might be of importance in the early post-exercise period in horses undertaking brief, high intensity fatiguing exercise. More research is needed to fully elucidate this finding, potentially utilizing additional measures such as protein catabolic markers (e.g., Akt and AMPK).

The metabolomic response seen in the conditioned state is likely due to the greater amount of work completed during acute exercise. Ultimately, fatigue is the final arbiter of running performance and it is well known that structured training improves running performance, in part through changes in skeletal muscle metabolism that increases the resistance to fatigue. Numerous hypotheses have been postulated in order to explain the cause of muscular fatigue in horses (Rivero and Piercy, 2014). These range from lactate production and the concomitant reduction in pH (Valberg, 1996); glycogen depletion during long-duration exercise (Snow et al., 1985); and cellular ion imbalances (Valberg, 2014). Another attractive hypothesis is the depletion of the intramuscular nucleotide pool that allows for the production and re-synthesis of nucleotides and ATP (Rivero and Piercy, 2014; Valberg, 2014). Indeed, ATP concentrations have been shown to decline following anaerobic, exhaustive exercise in skeletal muscle of the horse (Harris et al., 1991). As ATP concentrations decline, IMP concentrations increase due to high AMP-deaminase activity (Essen-Gustavsson et al., 1997). This ultimately slows the re-synthesis of ATP needed to sustain muscle contraction and promotes muscle fatigue. Therefore, IMP is consistently used a marker of nucleotide depletion in skeletal muscle of horses, the restoration of which may take up to an hour following exercise (Dunnett and Harris, 1999). The present findings showed that a variety of nucleotide intermediates were significantly elevated post-training, whereas IMP was significantly decreased. This suggests a beneficial augmentation of the nucleotide-related pool in conditioned skeletal muscle that could beneficially impact exercise tolerance. As demonstrated, the horses in the present study performed a greater amount of work and exercised for longer at higher exercise intensities following the training period. Thus, chronic training alters skeletal muscle nucleotide metabolism that, in part, may contribute to fatigue resistance. Additional direct testing is needed in order to confirm this relationship.

In humans and horses, endurance training increases aerobic and exercise capacities (Tyler et al., 1998; Scribbans et al., 2016), and improves insulin sensitivity and metabolic health (Powell et al., 2002; Goodpaster and Sparks, 2017). While no significant changes were seen in body composition over the training period, the horses exemplified body conditions and aerobic capacities similar to highly trained Standardbred racing horses (Kearns et al., 2002a, b). Intriguingly, there is evidence to suggest that the skeletal muscle phenotype in highly endurance-trained humans also closely mirrors a muscle phenotype that is seen in and/or is associated with obesity and insulin resistance (Goodpaster et al., 2001; Amati et al., 2011). This apparent inconsistency is aptly termed, “The Athlete’s Paradox” (Goodpaster et al., 2001). Notably, obesity and insulin resistance are associated with increased intramyocellular lipid (IMCL) (Goodpaster et al., 2001), as well as short-chain acylcarnitines (Koves et al., 2008; Baker et al., 2015), and increased blood concentrations of the BCAAs, phenylalanine and tyrosine (Felig et al., 1969; Newgard et al., 2009; Wang et al., 2011). Horses, like humans, also present with obesity and insulin resistance (Morgan et al., 2015) and are suggested to be a naturally occurring model for metabolic syndrome (Waller et al., 2011; Johnson et al., 2012). Interestingly, the present study showed an increase in most markers associated with obesity and insulin resistance (e.g., increased muscle DAGs and acylcarnitines, and increased blood BCAAs, phenylalanine and tyrosine) following the training period, suggesting that these metabolites may not be invariably linked to metabolic dysfunction. The extent to which this signature is evidence of paradoxical metabolism in horses remains unknown and more research is warranted.

It was also demonstrated that most lipid species, including certain DAGs, acylcarnitines, and almost every identified free fatty acid were significantly reduced by 24 h post-exercise in the conditioned state. Coupled with the altered expression of genes related to lipid uptake (*Cd36*) and utilization (*Cpt1b*), the current findings suggest that acute high-intensity exercise in the conditioned state limits the duration of time that potentially lipotoxic intermediates are present in skeletal muscle, possibly through net increased lipid utilization. While these findings are in agreement with the observation that IMTG can be significantly reduced at 24 and 48 h post-exercise (van Loon et al., 2003) to the best of our knowledge, no studies have directly evaluated the impact of acute high-intensity exercise on a wide variety of lipid species, such as DAGs or acylcarnitines, in mammalian skeletal muscle. These findings illustrate a strong gap in the literature regarding complex lipid species and their dynamics in muscle post-exercise.

Currently, there is limited and equivocal research addressing whether or not chronic exercise also alters resting plasma concentrations of amino acids, specifically the BCAAs, phenylalanine and tyrosine (Holm et al., 1978; Henriksson, 1991; Margolis et al., 2012). Indeed some studies have shown increases (Einspahr and Tharp, 1989; Margolis et al., 2012), no change (Holm et al., 1978), and even decreases (Pikosky et al., 2006) in plasma BCAAs with improved training status in humans. In horses, published information is limited to one longitudinal study examining the impact of training on amino acid concentrations (Westermann et al., 2011). In that study, 32 weeks of training did not alter the plasma concentrations of the individual BCAAs, phenylalanine or tyrosine. Conversely, in the present study, there was an increase in the concentrations of individual and total plasma BCAAs, phenylalanine, and tyrosine following the 12-week training period. This is in partial agreement with a 1989 study by Einspahr and Tharp (1989) that observed higher leucine, isoleucine, and tyrosine concentrations (valine was not measured) in trained *versus* relatively untrained high school runners, as well as another study by Margolis et al. showing increased total BCAAs in women following US Army basic combat training (Margolis et al., 2012). To the best of our knowledge, this is the first longitudinal study to report that training increases plasma BCAAs, phenylalanine and tyrosine in horses. This could be speculated to provide a readily available pool of amino acids for energy utilization and/or muscle recovery in the highly trained state. Ultimately, more research is needed to resolve the relationship between plasma BCAAs, aromatic amino acids, and training status in athletic mammals.

Another interesting observation was the presence of potentially microbially derived metabolites (Brown and Hazen, 2017) in skeletal muscle, particularly in the conditioned state. Previous results using the same horses have shown that 12 weeks of training significantly affects the gut microbiome (Janabi et al., 2016). Concomitant with changes in the gut microbiome, there was also a change in the skeletal muscle xenobiotic signature. This is suggestive of a gut-skeletal muscle axis (Grosicki et al., 2018). Interestingly, most xenobiotic or potentially microbially derived amino acid metabolites were increased to a greater extent 3 h post-exercise in conditioned *versus* unconditioned muscle. Evidence from human and equine exercise trials have shown the presence of an exercise-induced intestinal dysregulation, otherwise known as “leaky gut” (Baker et al., 1988; Donovan et al., 2007; Pires et al., 2017). These data preliminarily suggest the presence of a gut-skeletal muscle-axis in the horse that could be regulated, in part, via exercise-induced alterations in gut permeability. The extent to which this axis exists and modifies muscle metabolism or impacts performance in the horse warrants further investigation.

## Conclusion

The current study demonstrates the utility of untargeted metabolomics for the investigation into skeletal muscle metabolism with exercise, as well as highlights novel metabolic signatures related to the microbiome and amino acid, lipid, and nucleotide metabolism following training in the horse. Further, and perhaps most importantly, these results underscore the limitations of static metabolic measurements and the complexity of certain metabolites and their relationships in exercise, health and disease. Future studies using metabolomics-based approaches should couple these methods with other sophisticated techniques such as stable isotope metabolic tracer methodologies alongside transcriptomic and proteomic analyses in horses. A marriage of these methods will allow for a better understanding of metabolic fluxes as well as the gene-protein-metabolite networks and their influence on skeletal muscle metabolism.

## Data Availability Statement

All datasets generated for this study are included in the article/Supplementary Material.

## Ethics Statement

The animal study was reviewed and approved by The Rutgers University Institutional Animal Care and Use Committee (IACUC); Rutgers University.

## Author Contributions

DK, TA, and KM were involved in conception and design of the research, and interpreted results of the experiments. DK, EM, TA, and KM performed the experiments, edited and revised the manuscript, and approved final version of the manuscript. DK analyzed the data, prepared the figures, and drafted the manuscript.

## Conflict of Interest

The authors declare that the research was conducted in the absence of any commercial or financial relationships that could be construed as a potential conflict of interest.

## Footnotes

1 http://bioinfo.ut.ee/primer3-0.4.0/

2 https://assets.fishersci.com/tfs-assets/lsg/manuals/cms_046736.pdf

3 https://CRAN.R-project.org/package=org

## References

[B1] Amati F. Dube J. J. Alvarez-Carnero E. Edreira M. M. Chomentowski P. Coen P. M. (2011). Skeletal muscle triglycerides, diacylglycerols, and ceramides in insulin resistance: another paradox in endurance-trained athletes? *Diabetes* 60 2588–2597. 10.2337/db10-1221 21873552 PMC3178290

[B2] Baker B. Gaffin S. L. Wells M. Wessels B. C. Brock-Utne J. G. (1988). Endotoxaemia in racehorses following exertion. *J. S. Afr. Vet. Assoc.* 59 63–66. 3392702

[B3] Baker P. R. II Boyle K. E. Koves T. R. Ilkayeva O. R. Muoio D. M. Houmard J. A. (2015). Metabolomic analysis reveals altered skeletal muscle amino acid and fatty acid handling in obese humans. *Obesity* 23 981–988. 10.1002/oby.21046 25864501 PMC4414721

[B4] Bouwman F. G. van Ginneken M. M. Noben J. P. Royackers E. de Graaf-Roelfsema E. Wijnberg I. D. (2010). Differential expression of equine muscle biopsy proteins during normal training and intensified training in young standardbred horses using proteomics technology. *Comp. Biochem. Physiol. Part D Genomics Proteomics* 5 55–64. 10.1016/j.cbd.2009.11.001 20374942

[B5] Brown J. M. Hazen S. L. (2017). Targeting of microbe-derived metabolites to improve human health: the next frontier for drug discovery. *J. Biol. Chem.* 292 8560–8568. 10.1074/jbc.R116.765388 28389555 PMC5448085

[B6] Donovan D. C. Jackson C. A. Colahan P. T. Norton N. Hurley D. J. (2007). Exercise-induced alterations in pro-inflammatory cytokines and prostaglandin F2alpha in horses. *Vet. Immunol. Immunopathol.* 118 263–269. 10.1016/j.vetimm.2007.05.015 17617470

[B7] Dube J. J. Amati F. Stefanovic-Racic M. Toledo F. G. Sauers S. E. Goodpaster B. H. (2008). Exercise-induced alterations in intramyocellular lipids and insulin resistance: the athlete’s paradox revisited. *Am. J. Physiol. Endocrinol. Metab.* 294 E882–E888. 10.1152/ajpendo.00769.2007 18319352 PMC3804891

[B8] Dunnett M. Harris R. C. (1999). Influence of oral β-alanine and L-histidine supplementation on the carnosine content of the *gluteus medius*. *Equine Vet. J. Suppl.* 30 499–504. 10.1111/j.2042-3306.1999.tb05273.x 10659307

[B9] Einspahr K. J. Tharp G. (1989). Influence of endurance training on plasma amino acid concentrations in humans at rest and after intense exercise. *Int. J. Sports Med.* 10 233–236. 10.1055/s-2007-1024908 2606590

[B10] Eivers S. S. McGivney B. A. Fonseca R. G. MacHugh D. E. Menson K. Park S. D. (2010). Alterations in oxidative gene expression in equine skeletal muscle following exercise and training. *Physiol. Genomics* 40 83–93. 10.1152/physiolgenomics.00041.2009 19861432

[B11] Elder G. C. Bradbury K. Roberts R. (1982). Variability of fiber type distributions within human muscles. *J. Appl. Physiol. Respir. Environ. Exerc. Physiol.* 53 1473–1480. 10.1152/jappl.1982.53.6.1473 6218151

[B12] Essen-Gustavsson B. Roneus N. Poso A. R. (1997). Metabolic response in skeletal muscle fibres of standardbred trotters after racing. *Comp. Biochem. Physiol. B Biochem. Mol. Biol.* 117 431–436. 10.1016/s0305-0491(97)00140-5 9253181

[B13] Felig P. Marliss E. Cahill G. F. Jr. (1969). Plasma amino acid levels and insulin secretion in obesity. *N. Engl. J. Med.* 281 811–816. 10.1056/NEJM196910092811503 5809519

[B14] Gall W. E. Beebe K. Lawton K. A. Adam K. P. Mitchell M. W. Nakhle P. J. (2010). alpha-hydroxybutyrate is an early biomarker of insulin resistance and glucose intolerance in a nondiabetic population. *PLoS One* 5:e10883. 10.1371/journal.pone.0010883 20526369 PMC2878333

[B15] Goldansaz S. A. Guo A. C. Sajed T. Steele M. A. Plastow G. S. Wishart D. S. (2017). Livestock metabolomics and the livestock metabolome: a systematic review. *PLoS One* 12:e0177675. 10.1371/journal.pone.0177675 28531195 PMC5439675

[B16] Goodpaster B. H. He J. Watkins S. Kelley D. E. (2001). Skeletal muscle lipid content and insulin resistance: evidence for a paradox in endurance-trained athletes. *J. Clin. Endocrinol. Metab.* 86 5755–5761. 10.1210/jcem.86.12.8075 11739435

[B17] Goodpaster B. H. Sparks L. M. (2017). Metabolic flexibility in health and disease. *Cell Metab.* 25 1027–1036. 10.1016/j.cmet.2017.04.015 28467922 PMC5513193

[B18] Grosicki G. J. Fielding R. A. Lustgarten M. S. (2018). Gut microbiota contribute to age-related changes in skeletal muscle size, composition, and function: biological basis for a gut-muscle axis. *Calcif. Tissue Int.* 102 433–442. 10.1007/s00223-017-0345-345 29058056 PMC5858871

[B19] Harris R. C. Marlin D. J. Snow D. H. Harkness R. A. (1991). Muscle ATP loss and lactate accumulation at different work intensities in the exercising thoroughbred horse. *Eur. J. Appl. Physiol. Occup. Physiol.* 62 235–244. 10.1007/bf00571546 2044532

[B20] Heaney L. M. Deighton K. Suzuki T. (2017). Non-targeted metabolomics in sport and exercise science. *J. Sports Sci.* 37 959–967. 10.1080/02640414.2017.1305122 28346122

[B21] Henriksson J. (1991). Effect of exercise on amino acid concentrations in skeletal muscle and plasma. *J. Exp. Biol.* 160 149–165. 1960512 10.1242/jeb.160.1.149

[B22] Heyman E. Gamelin F. X. Aucouturier J. Di Marzo V. (2012). The role of the endocannabinoid system in skeletal muscle and metabolic adaptations to exercise: potential implications for the treatment of obesity. *Obes. Rev.* 13 1110–1124. 10.1111/j.1467-789X.2012.01026.x 22943701

[B23] Hodgson D. R. Foreman J. H. (2014). “CHAPTER 2 - Comparative aspects of exercise physiology,” in *The Athletic Horse*, 2nd Edn, eds Hodgson D. R. McKeever K. H. McGowan C. M. (Philadelphia PA: W.B. Saunders), 9–18. 10.1016/b978-0-7216-0075-8.00011-3

[B24] Hodgson D. R. Rose R. J. Dimauro J. Allen J. R. (1986). Effects of training on muscle composition in horses. *Am. J. Vet. Res.* 47 12–15. 3946889

[B25] Holm G. Sullivan L. Jagenburg R. Bjorntorp P. (1978). Effects of physical training and lean body mass of plasma amino acids in man. *J. Appl. Physiol. Respir. Environ. Exerc. Physiol.* 45 177–181. 10.1152/jappl.1978.45.2.177 681202

[B26] Itani S. I. Ruderman N. B. Schmieder F. Boden G. (2002). Lipid-induced insulin resistance in human muscle is associated with changes in diacylglycerol, protein kinase C, and IkappaB-alpha. *Diabetes* 51 2005–2011. 10.2337/diabetes.51.7.2005 12086926

[B27] Janabi A. Biddle A. Klein D. McKeever K. (2016). Exercise training-induced changes in the gut microbiota of Standardbred racehorses. *Comp. Exerc. Physiol.* 12 119–129.

[B28] Jang H. J. Kim D. M. Kim K. B. Park J. W. Choi J. Y. Oh J. H. (2017). Analysis of metabolomic patterns in thoroughbreds before and after exercise. *Asian-Australas J. Anim. Sci.* 30 1633–1642. 10.5713/ajas.17.0167 28728374 PMC5666199

[B29] Johnson P. J. Wiedmeyer C. E. LaCarrubba A. Ganjam V. K. Messer N. T. T. (2012). Diabetes, insulin resistance, and metabolic syndrome in horses. *J. Diabetes Sci. Technol.* 6 534–540. 10.1177/193229681200600307 22768883 PMC3440056

[B30] Kane R. A. Fisher M. Parrett D. Lawrence L. M. (1987). “Proc 10th Equine Nutr Physiol Symp,” in *Estimating fatness in horses*, (Ft. Collins, CO: Colorado State Univ).

[B31] Kearns C. F. McKeever K. H. (2002). Clenbuterol diminishes aerobic performance in horses. *Med. Sci. Sports Exerc.* 34 1976–1985. 10.1249/01.MSS.0000038973.96796.1E 12471305

[B32] Kearns C. F. McKeever K. H. Abe T. (2002a). Overview of horse body composition and muscle architecture: implications for performance. *Vet. J.* 164 224–234. 10.1053/tvjl.2001.0702 12505395

[B33] Kearns C. F. McKeever K. H. Kumagai K. Abe T. (2002b). Fat-free mass is related to one-mile race performance in elite standardbred horses. *Vet. J.* 163 260–266. 10.1053/tvjl.2001.0656 12090768

[B34] Klein D. J. Anthony T. G. McKeever K. M. (2020). Changes in maximal aerobic capacity, body composition, and running capacity with prolonged training and detraining in Standardbred horses. *Comp. Exerc. Physiol.* 1–10. (in press).

[B35] Koves T. R. Ussher J. R. Noland R. C. Slentz D. Mosedale M. Ilkayeva O. (2008). Mitochondrial overload and incomplete fatty acid oxidation contribute to skeletal muscle insulin resistance. *Cell Metab.* 7 45–56. 10.1016/j.cmet.2007.10.013 18177724

[B36] Lacombe V. A. Hinchcliff K. W. Geor R. J. Baskin C. R. (2001). Muscle glycogen depletion and subsequent replenishment affect anaerobic capacity of horses. *J. Appl. Physiol.* 91 1782–1790. 10.1152/jappl.2001.91.4.1782 11568163

[B37] Lacombe V. A. Hinchcliff K. W. Taylor L. E. (2003). Interactions of substrate availability, exercise performance, and nutrition with muscle glycogen metabolism in horses. *J. Am. Vet. Med. Assoc.* 223 1576–1585. 10.2460/javma.2003.223.1576 14664443

[B38] Lindholm A. Piehl K. (1974). Fibre composition, enzyme activity and concentrations of metabolites and electrolytes in muscles of standardbred horses. *Acta Vet. Scand.* 15 287–309. 4137664 10.1186/BF03547460 PMC8407315

[B39] Liu X. Locasale J. W. (2017). Metabolomics: a primer. *Trends Biochem. Sci.* 42 274–284. 10.1016/j.tibs.2017.01.004 28196646 PMC5376220

[B40] Lopez-Rivero J. L. Morales-Lopez J. L. Galisteo A. M. Aguera E. (1991). Muscle fibre type composition in untrained and endurance-trained Andalusian and Arab horses. *Equine Vet. J.* 23 91–93. 10.1111/j.2042-3306.1991.tb02727.x 2044515

[B41] Lopez-Rivero J. L. Serrano A. L. Diz A. M. Galisteo A. M. (1992). Variability of muscle fibre composition and fibre size in the horse gluteus medius: an enzyme-histochemical and morphometric study. *J. Anat.* 181 (Pt 1), 1–10. 1284127 PMC1259747

[B42] Margolis L. M. Pasiakos S. M. Karl J. P. Rood J. C. Cable S. J. Williams K. W. (2012). Differential effects of military training on fat-free mass and plasma amino acid adaptations in men and women. *Nutrients* 4 2035–2046. 10.3390/nu4122035 23250145 PMC3546621

[B43] McGivney B. A. Eivers S. S. MacHugh D. E. MacLeod J. N. O’Gorman G. M. Park S. D. (2009). Transcriptional adaptations following exercise in thoroughbred horse skeletal muscle highlights molecular mechanisms that lead to muscle hypertrophy. *BMC Genomics* 10:638. 10.1186/1471-2164-10-638 20042072 PMC2812474

[B44] McGivney B. A. McGettigan P. A. Browne J. A. Evans A. C. Fonseca R. G. Loftus B. J. (2010). Characterization of the equine skeletal muscle transcriptome identifies novel functional responses to exercise training. *BMC Genomics* 11:398. 10.1186/1471-2164-11-398 20573200 PMC2900271

[B45] McGowan C. M. Golland L. C. Evans D. L. Hodgson D. R. Rose R. J. (2002). Effects of prolonged training, overtraining and detraining on skeletal muscle metabolites and enzymes. *Equine Vet. J. Suppl.* 34 257–263. 10.1111/j.2042-3306.2002.tb05429.x 12405697

[B46] McKeever K. H. Malinowski K. (1997). Exercise capacity in young and old mares. *Am. J. Vet. Res.* 58 1468–1472. 9401701

[B47] Morgan R. Keen J. McGowan C. (2015). Equine metabolic syndrome. *Vet. Rec.* 177 173–179. 10.1136/vr.103226 26273009 PMC4552932

[B48] Newgard C. B. An J. Bain J. R. Muehlbauer M. J. Stevens R. D. Lien L. F. (2009). A branched-chain amino acid-related metabolic signature that differentiates obese and lean humans and contributes to insulin resistance. *Cell Metab.* 9 311–326. 10.1016/j.cmet.2009.02.002 19356713 PMC3640280

[B49] Pikosky M. A. Gaine P. C. Martin W. F. Grabarz K. C. Ferrando A. A. Wolfe R. R. (2006). Aerobic exercise training increases skeletal muscle protein turnover in healthy adults at rest. *J. Nutr.* 136 379–383. 10.1093/jn/136.2.379 16424115

[B50] Pires W. Veneroso C. E. Wanner S. P. Pacheco D. A. S. Vaz G. C. Amorim F. T. (2017). Association between exercise-induced hyperthermia and intestinal permeability: a systematic review. *Sports Med.* 47 1389–1403. 10.1007/s40279-016-0654-652 27943148

[B51] Poso A. R. Essen-Gustavsson B. Lindholm A. Persson S. G. B. (1991). Exercise-induced changes in muscle and plasma amino acid levels in the Standardbred horse. *Equine Exerc. Physiol.* 3 202–208.

[B52] Powell D. M. Reedy S. E. Sessions D. R. Fitzgerald B. P. (2002). Effect of short-term exercise training on insulin sensitivity in obese and lean mares. *Equine Vet. J. Suppl.* 34 81–84. 10.1111/j.2042-3306.2002.tb05396.x 12405664

[B53] Rivero J.-L. L. Piercy R. J. (2014). “6 - Muscle physiology: responses to exercise and training. *Equine Sports Med. Surg.* 2014 69–108.

[B54] Rodriguez L. P. Lopez-Rego J. Calbet J. A. Valero R. Varela E. Ponce J. (2002). Effects of training status on fibers of the musculus vastus lateralis in professional road cyclists. *Am. J. Phys. Med. Rehabil.* 81 651–660. 10.1097/00002060-200209000-200209004 12172517

[B55] Schuback K. Essen-Gustavsson B. Persson S. G. (1999). Incremental treadmill exercise until onset of fatigue and its relationship to metabolic response and locomotion pattern. *Equine Vet. J. Suppl.* 30 337–341. 10.1111/j.2042-3306.1999.tb05245.x 10659279

[B56] Scribbans T. D. Vecsey S. Hankinson P. B. Foster W. S. Gurd B. J. (2016). The effect of training intensity on vo2max in young healthy adults: a meta-regression and meta-analysis. *Int. J. Exerc. Sci.* 9 230–247. 27182424 10.70252/HHBR9374 PMC4836566

[B57] Snow D. H. Baxter P. Rose R. J. (1981). Muscle fibre composition and glycogen depletion in horses competing in an endurance ride. *Vet. Rec.* 108 374–378. 10.1136/vr.108.17.374 7292903

[B58] Snow D. H. Harris R. C. Gash S. P. (1985). Metabolic response of equine muscle to intermittent maximal exercise. *J. Appl. Physiol.* 58 1689–1697. 10.1152/jappl.1985.58.5.1689 3997731

[B59] Snow D. H. Mackenzie G. (1977). Effect of training on some metabolic changes associated with submaximal endurance exercise in the horse. *Equine Vet. J.* 9 226–230. 10.1111/j.2042-3306.1977.tb04037.x 923557

[B60] Storey J. D. Tibshirani R. (2003). Statistical significance for genomewide studies. *Proc. Natl. Acad. Sci. U.S.A.* 100 9440–9445. 10.1073/pnas.1530509100 12883005 PMC170937

[B61] Tyler C. M. Golland L. C. Evans D. L. Hodgson D. R. Rose R. J. (1998). Skeletal muscle adaptations to prolonged training, overtraining and detraining in horses. *Pflugers Arch.* 436 391–397. 10.1007/s004240050648 9644221

[B62] Valberg S. J. (1996). Muscular causes of exercise intolerance in horses. *Vet. Clin. North Am. Equine Pract.* 12 495–515. 10.1016/s0749-0739(17)30269-9 8938958

[B63] Valberg S. J. (2014). “CHAPTER 12 - Muscle anatomy, physiology, and adaptations to exercise and training,” in *The Athletic Horse*, 2nd Edn, eds Hodgson D. R. McKeever K. H. McGowan C. M. (St Louis, MO: Elsevier), 174–201. 10.1016/b978-0-7216-0075-8.00021-6

[B64] van Loon L. J. Schrauwen-Hinderling V. B. Koopman R. Wagenmakers A. J. Hesselink M. K. Schaart G. (2003). Influence of prolonged endurance cycling and recovery diet on intramuscular triglyceride content in trained males. *Am. J. Physiol. Endocrinol. Metab.* 285 E804–E811. 10.1152/ajpendo.00112.2003 12783774

[B65] Wagenmakers A. J. Brookes J. H. Coakley J. H. Reilly T. Edwards R. H. (1989). Exercise-induced activation of the branched-chain 2-oxo acid dehydrogenase in human muscle. *Eur. J. Appl. Physiol. Occup. Physiol.* 59 159–167. 10.1007/bf02386181 2583157

[B66] Wagner A. L. Urschel K. L. Betancourt A. Adams A. A. Horohov D. W. (2013). Effects of advanced age on whole-body protein synthesis and skeletal muscle mechanistic target of rapamycin signaling in horses. *Am. J. Vet. Res.* 74 1433–1442. 10.2460/ajvr.74.11.1433 24168310

[B67] Waller A. P. Burns T. A. Mudge M. C. Belknap J. K. Lacombe V. A. (2011). Insulin resistance selectively alters cell-surface glucose transporters but not their total protein expression in equine skeletal muscle. *J Vet Intern Med* 25 315–321. 10.1111/j.1939-1676.2010.0674.x 21314720

[B68] Waller A. P. Lindinger M. I. (2010). Nutritional aspects of post exercise skeletal muscle glycogen synthesis in horses: a comparative review. *Equine Vet. J.* 42 274–281. 10.2746/042516409X479603 20486986

[B69] Wang T. J. Larson M. G. Vasan R. S. Cheng S. Rhee E. P. McCabe E. (2011). Metabolite profiles and the risk of developing diabetes. *Nat. Med.* 17 448–453. 10.1038/nm.2307 21423183 PMC3126616

[B70] Westermann C. M. Dorland L. Wijnberg I. D. de Sain-van der Velden M. G. van Breda E. Barneveld A. (2011). Amino acid profile during exercise and training in Standardbreds. *Res. Vet. Sci.* 91 144–149. 10.1016/j.rvsc.2010.08.010 20863542

[B71] Westervelt R. G. Stouffer J. R. Hintz H. F. Schryver H. F. (1976). Estimating fatness in horses and ponies. *J. Anim. Sci.* 43 781–785. 10.2527/jas1976.434781x

[B72] White T. P. Brooks G. A. (1981). [U-14C]glucose, -alanine, and -leucine oxidation in rats at rest and two intensities of running. *Am. J. Physiol.* 240 E155–E165. 10.1152/ajpendo.1981.240.2.E155 6781361

[B73] Wittwer M. Billeter R. Hoppeler H. Fluck M. (2004). Regulatory gene expression in skeletal muscle of highly endurance-trained humans. *Acta Physiol. Scand.* 180 217–227. 10.1046/j.0001-6772.2003.01242.x 14738480

[B74] Wolfe R. R. Goodenough R. D. Wolfe M. H. Royle G. T. Nadel E. R. (1982). Isotopic analysis of leucine and urea metabolism in exercising humans. *J. Appl. Physiol. Respir. Environ. Exerc. Physiol.* 52 458–466. 10.1152/jappl.1982.52.2.458 7061300

[B75] Zhao X. Han Q. Liu Y. Sun C. Gang X. Wang G. (2016). The relationship between branched-chain amino acid related metabolomic signature and insulin resistance: a systematic review. *J. Diabetes Res.* 2016:2794591. 10.1155/2016/2794591 27642608 PMC5014958

