# Supplementary material for: Metabolomic Response of Equine Skeletal Muscle to Acute Fatiguing Exercise and Training
Source: Front Physiol. 2020 Feb 18;11:110. doi: 10.3389/fphys.2020.00110 (PMC7040365; doi:10.3389/fphys.2020.00110)
Supplement: Supplementary file 1 [file Table_1.docx]

| **Week** | **Day** | **Group A** | **Group B** |
| --- | --- | --- | --- |
|  | *Sunday* | Paddock | Paddock |
| **1** | *Monday* | **Light:**  5 min walk 2m/s  15 min trot 4m/s  5 min walk 2m/s | **Light:**  5 min walk 2m/s  15 min trot 4m/s  5 min walk 2m/s |
|  | *Tuesday* | **Moderate:**  5 min walk 2m/s  10 min trot 4m/s  5 min trot 6m/s  5 min walk 2m/s | **Light:**  5 min walk 2m/s  15 min trot 4m/s  5 min walk 2m/s |
|  | *Wednesday* | **Light:**  5 min walk 2m/s  15 min trot 4m/s  5 min walk 2m/s | **Moderate:**  5 min walk 2m/s  10 min trot 4m/s  5 min trot 6m/s  5 min walk 2m/s |
|  | *Thursday* | **Heavy:**  GXT to volitional fatigue | **Light:**  5 min walk 2m/s  15 min trot 4m/s  5 min walk 2m/s |
|  | *Friday* | **Light:**  5 min walk 2m/s  15 min trot 4m/s  5 min walk 2m/s | **Heavy:**  GXT to volitional fatigue |
|  | *Saturday* | Paddock | Paddock |
|  |  |  |  |
| **2** | *Sunday* | Paddock | Paddock |
|  | *Monday* | **Light:**  5 min walk 2m/s  20 min trot 4m/s  5 min walk 2m/s | **Light:**  5 min walk 2m/s  20 min trot 4m/s  5 min walk 2m/s |
|  | *Tuesday* | **Moderate:**  5 min walk 2m/s  15 min trot 4m/s  5 min trot 6m/s  5 min walk 2m/s | **Light:**  5 min walk 2m/s  20 min trot 4m/s  5 min walk 2m/s |
|  | *Wednesday* | **Light:**  5 min walk 2m/s  20 min trot 4m/s  5 min walk 2m/s | **Moderate:**  5 min walk 2m/s  15 min trot 4m/s  5 min trot 6m/s  5 min walk 2m/s |
|  | *Thursday* | **Heavy:**  GXT to volitional fatigue | **Light:**  5 min walk 2m/s  20 min trot 4m/s  5 min walk 2m/s |
|  | *Friday* | **Light:**  5 min walk 2m/s  20 min trot 4m/s  5 min walk 2m/s | **Heavy:**  GXT to volitional fatigue |
|  | *Saturday* | Paddock | Paddock |

**Supplementary Table S1. Schedule of 12-week training sessions**

| **Week** | **Day** | **Group A** | **Group B** |
| --- | --- | --- | --- |
|  | *Sunday* | Paddock | Paddock |
| **3** | *Monday* | **Light:**  5 min walk 2m/s  20 min trot 4m/s  5 min walk 2m/s | **Light:**  5 min walk 2m/s  20 min trot 4m/s  5 min walk 2m/s |
|  | *Tuesday* | **Moderate:**  5 min walk 2m/s  15 min trot 4m/s  5 min trot 6m/s  5 min walk 2m/s | **Light:**  5 min walk 2m/s  20 min trot 4m/s  5 min walk 2m/s |
|  | *Wednesday* | **Light:**  5 min walk 2m/s  20 min trot 4m/s  5 min walk 2m/s | **Moderate:**  5 min walk 2m/s  15 min trot 4m/s  5 min trot 6m/s  5 min walk 2m/s |
|  | *Thursday* | **Heavy:**  GXT to volitional fatigue | **Light:**  5 min walk 2m/s  20 min trot 4m/s  5 min walk 2m/s |
|  | *Friday* | **Light:**  5 min walk 2m/s  20 min trot 4m/s  5 min walk 2m/s | **Heavy:**  GXT to volitional fatigue |
|  | *Saturday* | Paddock | Paddock |
|  |  |  |  |
| **4** | *Sunday* | Paddock | Paddock |
|  | *Monday* | **Light:**  5 min walk 2m/s  20 min trot 4m/s  5 min walk 2m/s | **Light:**  5 min walk 2m/s  20 min trot 4m/s  5 min walk 2m/s |
|  | *Tuesday* | **Moderate:**  5 min walk 2m/s  10 min trot 4m/s  10 min trot 6m/s  5 min walk 2m/s | **Light:**  5 min walk 2m/s  20 min trot 4m/s  5 min walk 2m/s |
|  | *Wednesday* | **Light:**  5 min walk 2m/s  20 min trot 4m/s  5 min walk 2m/s | **Moderate:**  5 min walk 2m/s  10 min trot 4m/s  10 min trot 6m/s  5 min walk 2m/s |
|  | *Thursday* | **Heavy:**  GXT to volitional fatigue | **Light:**  5 min walk 2m/s  20 min trot 4m/s  5 min walk 2m/s |
|  | *Friday* | **Light:**  5 min walk 2m/s  20 min trot 4m/s  5 min walk 2m/s | **Heavy:**  GXT to volitional fatigue |
|  | *Saturday* | Paddock | Paddock |

| **Week** | **Day** | **Group A** | **Group B** |
| --- | --- | --- | --- |
|  | *Sunday* | Paddock | Paddock |
| **5** | *Monday* | **Light:**  5 min walk 2m/s  25 min trot 4m/s  5 min walk 2m/s | **Light:**  5 min walk 2m/s  25 min trot 4m/s  5 min walk 2m/s |
|  | *Tuesday* | **Moderate:**  5 min walk 2m/s  15 min trot 4m/s  10 min trot 6m/s  5 min walk 2m/s | **Light:**  5 min walk 2m/s  25 min trot 4m/s  5 min walk 2m/s |
|  | *Wednesday* | **Light:**  5 min walk 2m/s  25 min trot 4m/s  5 min walk 2m/s | **Moderate:**  5 min walk 2m/s  15 min trot 4m/s  10 min trot 6m/s  5 min walk 2m/s |
|  | *Thursday* | **Heavy:**  GXT to volitional fatigue | **Light:**  5 min walk 2m/s  25 min trot 4m/s  5 min walk 2m/s |
|  | *Friday* | **Light:**  5 min walk 2m/s  25 min trot 4m/s  5 min walk 2m/s | **Heavy:**  GXT to volitional fatigue |
|  | *Saturday* | Paddock | Paddock |
|  |  |  |  |
| **6** | *Sunday* | Paddock | Paddock |
|  | *Monday* | **Light:**  5 min walk 2m/s  30 min trot 4m/s  5 min walk 2m/s | **Light:**  5 min walk 2m/s  30 min trot 4m/s  5 min walk 2m/s |
|  | *Tuesday* | **Moderate:**  5 min walk 2m/s  20 min trot 4m/s  10 min trot 6m/s  5 min walk 2m/s | **Light:**  5 min walk 2m/s  30 min trot 4m/s  5 min walk 2m/s |
|  | *Wednesday* | **Light:**  5 min walk 2m/s  30 min trot 4m/s  5 min walk 2m/s | **Moderate:**  5 min walk 2m/s  20 min trot 4m/s  10 min trot 6m/s  5 min walk 2m/s |
|  | *Thursday* | **Heavy:**  GXT to volitional fatigue | **Light:**  5 min walk 2m/s  30 min trot 4m/s  5 min walk 2m/s |
|  | *Friday* | **Light:**  5 min walk 2m/s  30 min trot 4m/s  5 min walk 2m/s | **Heavy:**  GXT to volitional fatigue |
|  | *Saturday* | Paddock | Paddock |

| **Week** | **Day** | **Group A** | **Group B** |
| --- | --- | --- | --- |
|  | *Sunday* | Paddock | Paddock |
| **7** | *Monday* | **Light:**  5 min walk 2m/s  30 min trot 4m/s  5 min walk 2m/s | **Light:**  5 min walk 2m/s  30 min trot 4m/s  5 min walk 2m/s |
|  | *Tuesday* | **Moderate:**  5 min walk 2m/s  15 min trot 4m/s  15 min trot 6m/s  5 min walk 2m/s | **Light:**  5 min walk 2m/s  30 min trot 4m/s  5 min walk 2m/s |
|  | *Wednesday* | **Light:**  5 min walk 2m/s  30 min trot 4m/s  5 min walk 2m/s | **Moderate:**  5 min walk 2m/s  15 min trot 4m/s  15 min trot 6m/s  5 min walk 2m/s |
|  | *Thursday* | **Heavy:**  GXT to volitional fatigue | **Light:**  5 min walk 2m/s  30 min trot 4m/s  5 min walk 2m/s |
|  | *Friday* | **Light:**  5 min walk 2m/s  30 min trot 4m/s  5 min walk 2m/s | **Heavy:**  GXT to volitional fatigue |
|  | *Saturday* | Paddock | Paddock |
|  |  |  |  |
| **8** | *Sunday* | Paddock | Paddock |
|  | *Monday* | **Light:**  5 min walk 2m/s  30 min trot 4m/s  5 min walk 2m/s | **Light:**  5 min walk 2m/s  30 min trot 4m/s  5 min walk 2m/s |
|  | *Tuesday* | **Moderate:**  5 min walk 2m/s  10 min trot 4m/s  20 min trot 6m/s  5 min walk 2m/s | **Light:**  5 min walk 2m/s  30 min trot 4m/s  5 min walk 2m/s |
|  | *Wednesday* | **Light:**  5 min walk 2m/s  30 min trot 4m/s  5 min walk 2m/s | **Moderate:**  5 min walk 2m/s  10 min trot 4m/s  20 min trot 6m/s  5 min walk 2m/s |
|  | *Thursday* | **Heavy:**  GXT to volitional fatigue | **Light:**  5 min walk 2m/s  30 min trot 4m/s  5 min walk 2m/s |
|  | *Friday* | **Light:**  5 min walk 2m/s  30 min trot 4m/s  5 min walk 2m/s | **Heavy:**  GXT to volitional fatigue |
|  | *Saturday* | Paddock | Paddock |

| **Week** | **Day** | **Group A** | **Group B** |
| --- | --- | --- | --- |
|  | *Sunday* | Paddock | Paddock |
| **9** | *Monday* | **Light:**  5 min walk 2m/s  35 min trot 4m/s  5 min walk 2m/s | **Light:**  5 min walk 2m/s  35 min trot 4m/s  5 min walk 2m/s |
|  | *Tuesday* | **Moderate:**  5 min walk 2m/s  15 min trot 4m/s  15 min trot 6m/s  5 min walk 2m/s | **Light:**  5 min walk 2m/s  35 min trot 4m/s  5 min walk 2m/s |
|  | *Wednesday* | **Light:**  5 min walk 2m/s  35 min trot 4m/s  5 min walk 2m/s | **Moderate:**  5 min walk 2m/s  15 min trot 4m/s  15 min trot 6m/s  5 min walk 2m/s |
|  | *Thursday* | **Heavy:**  GXT to volitional fatigue | **Light:**  5 min walk 2m/s  35 min trot 4m/s  5 min walk 2m/s |
|  | *Friday* | **Light:**  5 min walk 2m/s  35 min trot 4m/s  5 min walk 2m/s | **Heavy:**  GXT to volitional fatigue |
|  | *Saturday* | Paddock | Paddock |
|  |  |  |  |
| **10** | *Sunday* | Paddock | Paddock |
|  | *Monday* | **Light:**  5 min walk 2m/s  35 min trot 4m/s  10 min walk 2m/s | **Light:**  5 min walk 2m/s  35 min trot 4m/s  10 min walk 2m/s |
|  | *Tuesday* | **Moderate:**  5 min walk 2m/s  10 min trot 4m/s  20 min trot 6m/s  5 min walk 2m/s | **Light:**  5 min walk 2m/s  35 min trot 4m/s  10 min walk 2m/s |
|  | *Wednesday* | **Light:**  5 min walk 2m/s  35 min trot 4m/s  10 min walk 2m/s | **Moderate:**  5 min walk 2m/s  10 min trot 4m/s  20 min trot 6m/s  5 min walk 2m/s |
|  | *Thursday* | **Heavy:**  GXT to volitional fatigue | **Light:**  5 min walk 2m/s  35 min trot 4m/s  10 min walk 2m/s |
|  | *Friday* | **Light:**  5 min walk 2m/s  35 min trot 4m/s  10 min walk 2m/s | **Heavy:**  GXT to volitional fatigue |
|  | *Saturday* | Paddock | Paddock |

| **Week** | **Day** | **Group A** | **Group B** |
| --- | --- | --- | --- |
|  | *Sunday* | Paddock | Paddock |
| **11** | *Monday* | **Light:**  5 min walk 2m/s  35 min trot 4m/s  10 min walk 2m/s | **Light:**  5 min walk 2m/s  35 min trot 4m/s  10 min walk 2m/s |
|  | *Tuesday* | **Moderate:**  5 min walk 2m/s  10 min trot 4m/s  20 min trot 6m/s  5 min walk 2m/s | **Light:**  5 min walk 2m/s  35 min trot 4m/s  10 min walk 2m/s |
|  | *Wednesday* | **Light:**  5 min walk 2m/s  35 min trot 4m/s  10 min walk 2m/s | **Moderate:**  5 min walk 2m/s  10 min trot 4m/s  20 min trot 6m/s  5 min walk 2m/s |
|  | *Thursday* | **Heavy:**  GXT to volitional fatigue | **Light:**  5 min walk 2m/s  35 min trot 4m/s  10 min walk 2m/s |
|  | *Friday* | **Light:**  5 min walk 2m/s  35 min trot 4m/s  10 min walk 2m/s | **Heavy:**  GXT to volitional fatigue |
|  | *Saturday* | Paddock | Paddock |
|  |  |  |  |
| **12-14*** | *Sunday* | Paddock | Paddock |
|  | *Monday* | **Light:**  5 min walk 2m/s  35 min trot 4m/s  10 min walk 2m/s | **Light:**  5 min walk 2m/s  35 min trot 4m/s  10 min walk 2m/s |
|  | *Tuesday* | **Moderate:**  5 min walk 2m/s  10 min trot 4m/s  20 min trot 6m/s  5 min walk 2m/s | **Light:**  5 min walk 2m/s  35 min trot 4m/s  10 min walk 2m/s |
|  | *Wednesday* | **Light:**  5 min walk 2m/s  35 min trot 4m/s  10 min walk 2m/s | **Moderate:**  5 min walk 2m/s  10 min trot 4m/s  20 min trot 6m/s  5 min walk 2m/s |
|  | *Thursday* | **Heavy:**  GXT to volitional fatigue | **Light:**  5 min walk 2m/s  35 min trot 4m/s  10 min walk 2m/s |
|  | *Friday* | **Light:**  5 min walk 2m/s  35 min trot 4m/s  10 min walk 2m/s | **Heavy:**  GXT to volitional fatigue |
|  | *Saturday* | Paddock | Paddock |

*This training schedule was maintained for an additional two weeks during the cross-over period
