# Supplementary material for: Metabolomic Response of Equine Skeletal Muscle to Acute Fatiguing Exercise and Training
Source: Front Physiol. 2020 Feb 18;11:110. doi: 10.3389/fphys.2020.00110 (PMC7040365; doi:10.3389/fphys.2020.00110)
Supplement: Supplementary file 2 [file Table_2.pdf]

Supplementary Table S2. Table showing statistical comparisons by way of ANOVA contrasts in the unconditioned and conditioned states. From analysis of the dataset, a total 545 named biochemicals were detected. Red signifies the number of metabolites significantly ($p<0.05$; $q<0.1$) increased; green signifies the number of metabolites significantly ($p<0.05$; $q<0.1$) decreased.

Statistical Comparisons ANOVA Contrasts			
		**Total biochemicals ($p<0.05$; $q<0.1$)**	**Biochemicals (↑↓)**
**Unconditioned**	***T3* / *Pre***	31	29|2
	T24* / *Pre	1	1|0
	T24* / *T3	12	1|11
**Conditioned**	***T3* / *Pre***	142	100|42
	T24* / *Pre	150	13|137
	T24* / *T3	223	26|197
